# Intensity of Mystical Experiences Occasioned by 5-MeO-DMT and Comparison With a Prior Psilocybin Study

**DOI:** 10.3389/fpsyg.2018.02459

**Published:** 2018-12-06

**Authors:** Joseph Barsuglia, Alan K. Davis, Robert Palmer, Rafael Lancelotta, Austin-Marley Windham-Herman, Kristel Peterson, Martin Polanco, Robert Grant, Roland R. Griffiths

**Affiliations:** ^1^Crossroads Treatment Center, Tijuana, Mexico; ^2^Terra Incognita Project, NGO, Ben Lomond, CA, United States; ^3^New School Research, LLC, North Hollywood, CA, United States; ^4^The Mission Within, Oakland, CA, United States; ^5^Department of Psychiatry, Johns Hopkins University School of Medicine, Baltimore, MD, United States; ^6^Yale School of Medicine, New Haven, CT, United States; ^7^Department of Counseling, Leadership, Advocacy, and Design, University of Wyoming, Laramie, WY, United States; ^8^California Institute of Integral Studies, San Francisco, CA, United States; ^9^Gladstone Institutes, University of California, San Francisco, San Francisco, CA, United States; ^10^Department of Neuroscience, Johns Hopkins University School of Medicine, Baltimore, MD, United States

**Keywords:** 5-MeO-DMT, tryptamines, psychedelic, mystical experience, DMT, *Bufo alvarius*, Colorado river toad

## Abstract

5-MeO-DMT is a psychoactive substance found in high concentrations in the bufotoxin of the Colorado River Toad (*Bufo alvarius*). Emerging evidence suggests that vaporized 5-MeO-DMT may occasion mystical experiences of comparable intensity to those occasioned by more widely studied psychedelics such as psilocybin, but no empirical study has tested this hypothesis. Data was obtained from 20 individuals (*M*_age_ = 38.9, ± 10.7; male = 55%, Caucasian = 85%) who were administered 5-MeO-DMT as part of a psychospiritual retreat program in Mexico. All participants received 50 mg of inhaled vaporized toad bufotoxin which contains 5-MeO-DMT and completed the Mystical Experience Questionnaire (MEQ30) approximately 4–6 h after their session. Administration of 5-MeO-DMT occasioned strong mystical experiences (MEQ30 Overall *M*_intensity_ = 4.17, ± 0.64, range 0–5) and the majority (*n* = 15, 75%) had “a complete mystical experience” (≥60% on all MEQ30 subscales). Compared to a prior laboratory-based psilocybin study, there were no differences in the intensity of mystical effects between 5-MeO-DMT and a high dose (30 mg/70 kg) of psilocybin, but the intensity of mystical effects was significantly higher in the 5-MeO-DMT sample compared to moderate/high dose (20 mg/70 kg) of psilocybin (MEQ30 Total Score: *p* = 0.02, *d* = 0.81). Administration of vaporized 5-MeO-DMT reliably occasioned complete mystical experiences in 75% of individuals and was similar in intensity to high dose psilocybin administered in a laboratory setting. The short duration of action may be advantageous for clinical interventions and for studying mystical-type experiences.

## Introduction

5-Methoxy-*N,N*-dimethyltryptamine (5-MeO-DMT) is a psychoactive indolealkylamine (Yu, 2008; Szabo et al., 2014) that is present in the bufotoxin of the Colorado River toad (*Bufo alvarius*) (Weil and Davis, 1994; Lyttle et al., 1996), numerous plant species (Smith, 1977; Ott, 2001; Shulgin and Shulgin, 2002), and can be synthetically produced (Hoshino and Shimodaira, 1936). Preliminary evidence shows that 5-MeO-DMT occasions mystical experiences (Davis et al., in press) similar in intensity to those occasioned by psilocybin (Griffiths et al., 2011), with a much shorter duration of action (half-life 12–19 min; Shen et al., 2010). Mystical experiences occasioned by psilocybin are a primary predictor of therapeutic outcomes in patients with substance use disorders (Garcia-Romeu et al., 2014; Bogenschutz et al., 2015) and depression/anxiety (Cowen, 2016; Griffiths et al., 2016). Such experiences are reported as profoundly meaningful, spiritual and transformative peak life events, and are associated with adaptive outcomes across a range of psychological domains (Griffiths et al., 2018; Roseman et al., 2018). However, the lack of laboratory-based studies examining the dose-related effects of 5-MeO-DMT in humans limits our understanding of this substance. Furthermore, no published studies have directly compared the intensity of mystical experiences occasioned by 5-MeO-DMT to psilocybin administered in the laboratory.

The current study aimed to address this gap in the literature via two primary aims. The first aim was to examine the intensity of mystical experiences following administration of 5-MeO-DMT (in the form of vaporized bufotoxin) to participants in a residential psychospiritual retreat. We hypothesized that these participants would rate the intensity of mystical effects as moderate-to-strong, similar to the prior findings for 5-MeO-DMT users in a survey study (Davis et al., 2018; Davis et al., in press). The second aim was to compare the intensity of mystical experiences occasioned by 5-MeO-DMT in the present study with that recorded in a prior laboratory-based psilocybin study (Griffiths et al., 2011). We hypothesized that the intensity of mystical experiences would be similar to that reported by individuals administered high-dose psilocybin and greater than that reported by individuals administered a moderate/high dose of psilocybin.

## Materials and Methods

### Participants

All 20 participants (11 males) were residents of a psychospiritual retreat at a center in Baja California, Mexico, between August 2015 and May 2017. They were medically healthy as determined by medical examination and history, electrocardiography, and urine drug testing. Participants had an average age of 38 years (range, 21–57 years). Eight participants (40% of the sample) had a college degree, five (25%) had a graduate degree, and three (15%) had a high school diploma. Seventeen participants (85%) reported their ethnicity as White/Caucasian; one (5%) Asian; one (5%) Latino or Hispanic; and one (5%) other. Sixteen of the participants (80%) were from the United States; two (10%) from Canada; and two (10%) from Australia and New Zealand. Nine participants (45%) reported having a religious affiliation that included three (15%) affiliated with Christianity, two (10%) with inter/non-denominational faith, two (10%) with Hinduism, one (5%) with Judaism, and one (5%) with Buddhism.

### Procedures

At the center, all residents were accepted into the four-day retreat program on a fee-for-service basis that included administration of vaporized 5-MeO-DMT and therapeutic preparation, education, and integration. Each resident received 50 mg of vaporized bufotoxin, estimated to contain 5–7 mg of 5-MeO-DMT (5-MeO-DMT content of 10–15%, considered a “light” to “common” dose) (Weil and Davis, 1994; Metzner, 2013; Erowid, 2017). The bufotoxin was obtained from wild toads in the Sonoran Desert, Mexico. The bufotoxin was kept in a glass vial attachment of an Eclipse (Herbal, 2016) handheld vaporizer and heated with a torch lighter to the point of vaporization of all contents. As soon as vapors began to be emitted, residents were instructed to fully exhale and then slowly fill their lungs to capacity and hold the full inhalation for a minimum of 10s. At the end of 10s, the facilitator guided residents to exhale and lay down. A medical doctor and facilitator/guide were present during the session. Within 4–6 h following their experience, residents were given the option to complete the Mystical Experience Questionnaire (described below) as part of a brief anonymous exit program evaluation and satisfaction survey on an online survey platform^1^. Of the 76 residents who completed the exit survey, 20 opted to complete the questionnaire (26.3% completion rate). No identifiable data was collected and the survey was stored in a secured database separate from clinic records.

### Ethics Statement

The manuscript is based on a secondary analysis of previously collected data and the authors were only given access to anonymous and de-identified data. This retrospective analysis of archival data was determined exempt from review (IntegReview IRB, Austin TX) and informed consent was not required. The source of the original data was anonymous survey responses and was provided by Crossroads Treatment Center for analysis.

### Instruments

#### Mystical Effects

We used the 30 items of the Mystical Experience Questionnaire (MEQ30; Barrett et al., 2015) to measure acute mystical effects of 5-MeO-DMT. The MEQ30 was rescored from the 100-item States of Consciousness Questionnaire (SOCQ) (Griffiths et al., 2006). Participants rated the intensity with which they experienced each item on a 6-point scale [from “0 = none; not at all” to “5 = extreme (more than ever before in my life and stronger than 4)”] (Griffiths et al., 2006). MEQ30 total scores range from 0 to 150. The MEQ30 has four factors (mystical [which includes unitive experiences, noetic quality, and sacredness], positive mood, transcendence of time/space, and ineffability) described in Barrett et al. (2015). A participant was scored as having had a “complete mystical experience” when ≥60% of the maximum possible score was endorsed on all four MEQ subscales. The MEQ30 has demonstrated sensitivity in assessing the effects of a range of psychedelic compounds, including LSD (Schmid and Liechti, 2017), MDMA (Lyvers and Meester, 2012), psilocybin (Barrett et al., 2015), ayahuasca (Schenberg et al., 2017), and 5-MeO-DMT (Davis et al., 2018).

### Data Analysis

Demographic data were analyzed using frequency counts. Correlational analyses were used to determine significant associations between demographic values and MEQ30 scores. Independent sample *t*-tests were performed to compare the MEQ30 ratings for the current sample with those from a laboratory psilocybin study (Griffiths et al., 2011; Barrett et al., 2015; Appendix 3). Because of the limitations associated with using significance tests as a primary statistical procedure and the limitations of using a corrected alpha in exploratory studies, a standard alpha of 0.05 was used to determine the significance of statistical tests and effect sizes (Cohen’s *d*) were calculated for each test to assist with interpretation of meaningful effects.

## Results

### Acute Subjective Mystical Effects of 5-MeO-DMT

There were no significant correlations between MEQ30 scores and any demographic variables (data not presented but available upon request). Overall, 75% of the participants met the criteria for “a complete mystical experience” (scoring ≥60% of the maximum possible scores on all four MEQ subscales). Mean scores across the MEQ30 subscales ranged from 79% to 89% of the maximum possible score (See Table 1). Item means for each subscale were consistent with the rating of a “strong” mystical experience: mystical, 3.96 ± 0.92; positive mood, 4.43 ± 0.58; transcendence of time and space, 4.28 ± 0.69; ineffability, 4.43 ± 0.63; and the MEQ30 total score, 4.17 ± 0.64.

**Table 1:** MEQ30 data after 5-MeO-DMT administration in a psychospiritual sample compared to the findings obtained with moderate and high doses of psilocybin in a previously published study by Griffiths et al. (2011) (scores from Barrett et al., 2015).

	5-MeO-DMTEst. 5–7 mg (n = 20)	Psilocybin30 mg/70 kg (n = 18)	Psilocybin20 mg/70 kg (n = 18)
*MEQ Mystical*	*79.27 (4.11)*	*73.04 (6.19)*	*66.89 (5.72)*
*MEQ Positive Mood*	*88.67 (2.61)^∗∗^*	*79.26 (5.58)*	*72.22 (4.93)*
*MEQ Time/Space*	*85.67 (3.09)^∗^*	*79.63 (4.74)*	*69.07 (5.93)*
*MEQ Ineffability*	*88.67 (2.82)*	*85.19 (6.30)*	*78.52 (6.72)*
*Total MEQ Score*	*83.37 (2.88)^∗^*	*76.81 (5.28)*	*69.56 (5.04)*
*Complete Experience (%)*	*75%*	*67%*	*61%*

*The data show MEQ30 total and subscale means (% of maximum scores) and SEMs, and percentages of participants reporting “complete” mystical experiences. “Complete mystical experience” reports the percentage of participants at each dose level whose scores on all four of the MEQ30 factors were greater than or equal to 60% of the maximum possible score. There were no significant differences between the 5-MeO-DMT and 30 mg/70 kg Psilocybin group. Significant differences between the 5-MeO-DMT and 20 mg/70 kg group are identified as ^∗^*p* < 0.05, ^∗∗^*p* < 0.01*.

### Comparison of MEQ30 Scores With Ratings From a Prior Psilocybin Study

**FIGURE 1:**
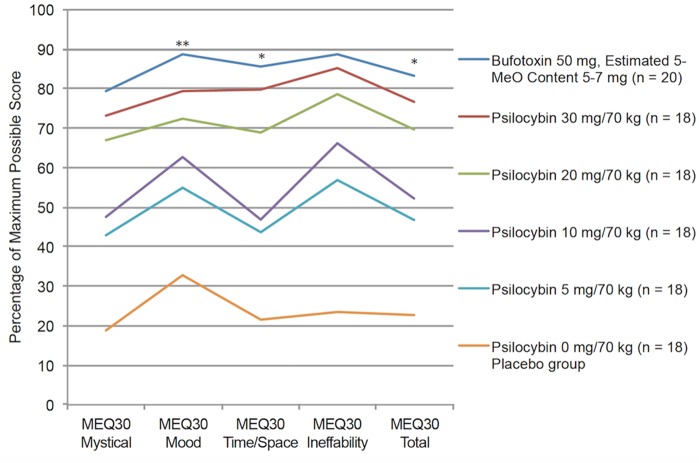
Comparison of psilocybin and 5-MeO-DMT groups on the Mystical Experiences Questionnaire (MEQ30). 5-MeO-DMT results from current study and psilocybin ratings from the Griffiths et al., 2011 dose-related effects study. No significant differences are observed between the 5-MeO-DMT and 30 mg/70 kg Psilocybin group. Significant differences are observed between the 5-MeO-DMT and 20 mg/70 kg group. ^∗^*p* < 0.05, ^∗∗^*p* < 0.01. The 10 mg/70 kg, 5 mg/70 kg, and 0 mg placebo groups from Griffiths et al. (2011) are plotted for visual reference and were not statistically compared.

MEQ30 ratings in the present study were compared to those previously reported by healthy volunteers (i.e., psychiatrically and medically healthy, hallucinogen naive) administered a moderate/high dose (20 mg/70 kg, *n* = 18) and a high dose (30 mg/70 kg, *n = 18*) of psilocybin (Griffiths et al., 2011) (See Table 1). As Table 1 and Figure 1 show, all MEQ30 ratings from the 5-MeO-DMT sample were statistically equivalent to MEQ30 ratings from the high-dose psilocybin sample (all *p*-values > 0.11). However, when compared to the moderate-high dose psilocybin sample, the MEQ30 ratings for the 5-MeO-DMT sample were significantly greater on the positive mood (*p* = 0.004, *d* = 1.01) and transcendence of time/space (*p* = 0.015, *d* = 0.85) subscales, and the total MEQ30 score (*p* = 0.02, *d* = 0.81). Additionally, the proportion of participants who received 5-MeO-DMT and reported a complete mystical experience was 75%, whereas the corresponding proportions for the psilocybin samples were 66.7% (30 mg/70 kg) and 61.1% (20 mg/70 kg), which were not significantly different (*p*-values > 0.36) (See Table 1 and Figure 1).

## Discussion

Several investigators have suggested that mystical-type experience occasioned by psychedelics predicts lasting psychiatric and behavioral changes and treatment efficacy (Griffiths et al., 2018; Roseman et al., 2018). Thus, this study aimed to examine the intensity of mystical experiences following administration of 5-MeO-DMT to participants as part of a residential psychospiritual retreat, and to compare the intensity of mystical experiences occasioned by 5-MeO-DMT with those recorded in a prior laboratory-based psilocybin study. Consistent with our hypothesis, participants in the retreat program rated the intensity of mystical effects occasioned by 5-MeO-DMT as moderate-to-strong, similar to prior findings in samples of 5-MeO-DMT users (Davis et al., 2018; Davis et al., in press). Moreover, the overall intensity of mystical effects occasioned by an estimated light to common dose of 5-MeO-DMT (5–7 mg) (Weil and Davis, 1994; Metzner, 2013; Erowid, 2017) was statistically equivalent to the ratings for a high-dose (30 mg/70 kg) psilocybin session and significantly higher than a moderate/high-dose (20 mg/70 kg) psilocybin session, as reported in a previous study of healthy volunteers (Griffiths et al., 2011, presented in Barrett et al., 2015).

Nevertheless, the current study had several limitations. First, this study was neither prospective nor placebo-controlled. Additionally, each dose of bufotoxin likely had slightly varying potencies across participants because 5-MeO-DMT concentrations vary across toad samples, and because bufotoxin contains relatively smaller concentrations of other tryptamines (Erspamer et al., 1967; Weil and Davis, 1994; Qi et al., 2018), which may alter the subjective experience in comparison to synthetically produced 5-MeO-DMT. Furthermore, participants received 5-MeO-DMT in a four-day program that included ibogaine administration 48 h before 5-MeO-DMT. The active metabolite of ibogaine, noribogaine, has a half-life of 28–49 h (Glue et al., 2015), and may potentiate 5-MeO-DMT experiences. The study is also limited by a lack of diverse representation, most of the samples were educated Caucasian individuals from the United States, limiting the generalizability of the findings. Moreover, extensive psychiatric history of the residents was not obtained; thus, we cannot rule out the possibility that some participants may not have been “healthy volunteers,” unlike the individuals in the psilocybin study.

5-MeO-DMT appears to have a relatively safe profile for use in naturalistic settings (Davis et al., in press) and in the general population (Davis et al., 2018) as evidenced by reports of low addiction liability (i.e., craving, legal, medical, psychiatric problems associated with consumption), which requires further investigation in a phase 1 trial. Initial evidence suggests 5-MeO-DMT shows possible psychotherapeutic effects, as individuals from a survey study (Davis et al., 2018; Davis et al., in press) who reported being diagnosed with depression, anxiety, post-traumatic stress disorder, and substance addictions reported improvement in their symptoms following 5-MeO-DMT use. The results of the present study extend the previous findings by demonstrating that the subjective mystical effects of 5-MeO-DMT administered in a retreat setting are similar to those reported in prior 5-MeO-DMT studies, and that the intensity of these effects is statistically equivalent to that of high-dose psilocybin administered in a laboratory setting. Since a light to common dose of 5-MeO-DMT appears to occasion mystical experiences of a comparatively similar intensity to high-dose psilocybin but with a much shorter duration of action, 5-MeO-DMT may have psychotherapeutic applications and could be relatively easier to use in mental health treatment systems. It is also a compound of interest for characterizing mystical-type experiences and their consequences. Since the intensity of mystical experiences occasioned by psychedelics may predict their therapeutic efficacy (Roseman et al., 2018), further research is warranted on the use of 5-MeO-DMT as a potential therapeutic agent in the treatment of multiple mental health issues.

## Author Contributions

JB was responsible for study conceptualization, study design, data collection, data analyses, and the initial draft of the manuscript and editing. AD was responsible for study conceptualization and the initial draft of the manuscript and editing. RRG was responsible for study conceptualization and manuscript editing. RL and KP was responsible for database organization and manuscript editing. MW-H, RP, MP, and RG were responsible for manuscript editing.

## Conflict of Interest Statement

RRG is a member of the Board of Directors of the Heffter Research Institute. The remaining authors declare that the research was conducted in the absence of any commercial or financial relationships that could be construed as a potential conflict of interest.

## Footnotes

1 http://www.SurveyMonkey.com
